# The *D4Z4* Macrosatellite Repeat Acts as a CTCF and A-Type Lamins-Dependent Insulator in Facio-Scapulo-Humeral Dystrophy

**DOI:** 10.1371/journal.pgen.1000394

**Published:** 2009-02-27

**Authors:** Alexandre Ottaviani, Sylvie Rival-Gervier, Amina Boussouar, Andrea M. Foerster, Delphine Rondier, Sabrina Sacconi, Claude Desnuelle, Eric Gilson, Frédérique Magdinier

**Affiliations:** 1 Laboratoire de Biologie Moléculaire de la Cellule, Ecole Normale Supérieure de Lyon, Centre National de la Recherche Scientifique UMR 5239, UCBL1, IFR128, Lyon, France; 2 Institut National de la Recherche Agronomique, ENVA, UMR 1198, Biologie du Développement et de la Reproduction, Centre National de la Recherche FRE 2857, Jouy-en-Josas, France; 3 Centre de Référence pour les Maladies Neuromusculaires, CHU de Nice, Nice, France; Netherlands Cancer Institute, Netherlands

## Abstract

Both genetic and epigenetic alterations contribute to Facio-Scapulo-Humeral Dystrophy (FSHD), which is linked to the shortening of the array of *D4Z4* repeats at the 4q35 locus. The consequence of this rearrangement remains enigmatic, but deletion of this 3.3-kb macrosatellite element might affect the expression of the FSHD-associated gene(s) through position effect mechanisms. We investigated this hypothesis by creating a large collection of constructs carrying 1 to >11 *D4Z4* repeats integrated into the human genome, either at random sites or proximal to a telomere, mimicking thereby the organization of the 4q35 locus. We show that *D4Z4* acts as an insulator that interferes with enhancer–promoter communication and protects transgenes from position effect. This last property depends on both CTCF and A-type Lamins. We further demonstrate that both anti-silencing activity of *D4Z4* and CTCF binding are lost upon multimerization of the repeat in cells from FSHD patients compared to control myoblasts from healthy individuals, suggesting that FSHD corresponds to a gain-of-function of CTCF at the residual *D4Z4* repeats. We propose that contraction of the *D4Z4* array contributes to FSHD physio-pathology by acting as a CTCF-dependent insulator in patients.

## Author Summary

Facio-Scapulo-Humeral Dystrophy (FSHD) is the third most common myopathy with an autosomal-dominant mode of inheritance. FSHD is caused by contraction of an array of repeated sequences, *D4Z4*, in the terminal region of chromosome 4 (4q35 locus). This genetic disease is not caused by classical mutations within the sequence of a gene but rather is associated with a change in the organization of the chromatin fiber. Because of the complexity of the region implicated in the disease, the exact pathogenic mechanism is still unclear. Our goal was to engineer genomic tools that would reproduce the organization of the chromosomal region linked to FSHD in order to understand the biological function of the *D4Z4* repeat using cellular models. We have identified a new mechanism for the regulation of the *D4Z4* array depending on both the number of repeats and the presence of CTCF and A-type Lamins. Our work reveals that D4Z4 acts as a potent insulator element that protects from the influence of repressive chromatin in patient cells but not in controls. Besides the importance of these findings for the understanding of this complex muscular dystrophy, our work also uncovers a new insulator element that regulates chromatin in human cells.

## Introduction

The subtelomeric regions that lie between the telomeres and the proximal gene-rich regions display a variable size distribution and contribute to genome evolution but also human disorders [1]. In the Facio-Scapulo-Humeral Dystrophy (FSHD), the contraction of an array of macrosatellite elements at the 4q35 locus is associated with pathological cases [2]. Normal 4q35 chromosome end carries 11 to up to 100–150 integral copies of the 3.3 kb *D4Z4* sequence while in FSHD patients, the pathogenic allele has only 1 to 10 repeats [3],[4]. This autosomal dominant disorder is the third most common myopathy, clinically described as a progressive and asymmetric weakening of the muscles of the face, scapular girdle and upper limbs [5]. The nature and function of the genes causing the pathology are still controversial [6],[7],[8]. Indeed, the pathogenic alteration does not reside within a specific gene but the FSHD-associated gene(s) might be rather regulated in *cis* or *trans* by chromatin modifications and epigenetic alterations linked to the number of repeats [9]. Several molecular mechanisms have been proposed to explain FSHD pathogenesis [9],[10] such as the implication of position effect variegation (PEV) or telomeric position effect (TPE) [9],[10]. However, these hypotheses have never been formally demonstrated.

*D4Z4* belongs to a family of repetitive DNA sequences present at different *loci* in the human genome including the 10qter, which is 98% homologous to the 4q35 region. The array of *D4Z4* on chromosome 10 is also polymorphic but is not associated with any disease [11],[12]. Intriguingly, the main difference between the 10qter and the 4q35 locus resides in their respective subnuclear positioning [13],[14] suggesting that the FSHD pathogenesis might result from inappropriate chromatin interactions [15] depending on the number of *D4Z4* elements in a particular subnuclear context.

In order to understand the molecular mechanisms leading to FSHD, we investigated the functional properties of the *D4Z4* subtelomeric repeat by engineering different cellular models that mimic the basic organization of the 4q35 locus. We found that a single *D4Z4* behaves as a potent insulator interfering with enhancer-promoter communication and shielding from chromosomal position effect (CPE). This last property depends upon CTCF and A-type Lamins. Intriguingly, both CTCF binding and insulation activity are lost upon multimerization of the repeats suggesting that FSHD results from an inappropriate insulation mechanism and a CTCF-gain of function. The implication for FSHD pathogenesis is discussed.

## Results

### *D4Z4* Behaves as an Insulator Element

**Figure 1:**
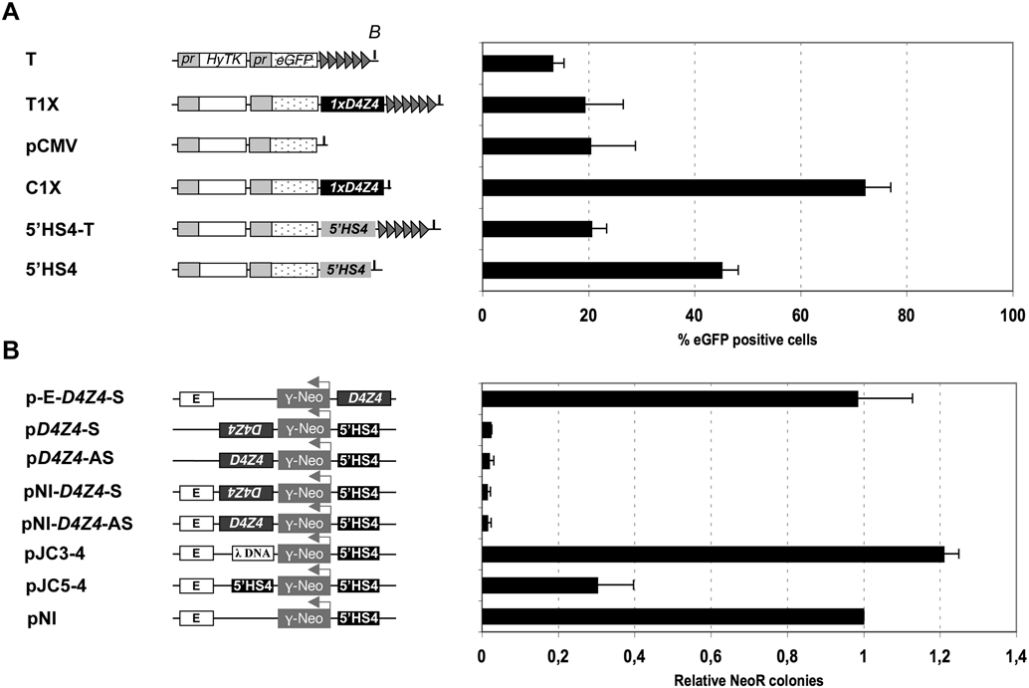
A single *D4Z4* acts as a boundary that interferes with position effect and enhancer-promoter communication. A. The different constructs carry a hygromycin resistance gene fused to the herpes simplex virus type 1 thymidine kinase suicide gene (*HyTK*, white box) and an *eGFP* reporter gene (speckled box), each driven by a CMV promoter (pr). In the T construct, a telomere seed (grey triangles) is added downstream of the *eGFP* reporter gene in order to create a *de novo* telomere after random integration followed by a telomeric fragmentation [47]. A single *D4Z4* repeat (black box) is cloned downstream of the *eGFP* gene in pCMV construct (C1X) or between *eGFP* and the telomere seed in T construct (T1X). We further compared *D4Z4* with the canonical chicken *5′ HS4* boundary [19] by cloning this latest sequence into the vectors used for *de novo* telomere seeding (5′HS4-T) or for random integration (5′HS4). Each constructs were linearized and transfected into the human cervical carcinoma cells (C33A). The level of eGFP was measured by flow cytometry (FACS) for an extended period of time in the presence or absence of Hygromycin B (Figure S1A). Histograms show the average percentage of eGFP positive cells from day 18 to day 29 of three independent transfections ±S.D. shown by error bars, when *eGFP* expression reaches a plateau (Figure S1B). The integrity of each construct was verified in stable populations of cells (Figure S1D). B. In order to evaluate the enhancer blocking activity of *D4Z4*, we used the test previously described [19]. The K562 human erythroleukemia cell line was stably transfected with the constructs shown on the left. Each construct carries the neomycin resistance gene driven by the human A *β*-globin promoter (*γ*-Neo) flanked with the mouse *5′HS2* enhancer (E). Most constructs contain the *5′HS4* insulator upstream of the promoter in order to block from the influence of regulatory elements at the site of integration. For each assay, colony number was normalized to the un-insulated control (pNI). Data are the average of three independent transfections. The mean values with S.D. are plotted. As controls, the following constructs, kindly provided by Dr. G. Felsenfeld, were used: pNI, no insert; pJC3-4, 2.3 kb of λ DNA; pJC5-4, chicken *β-globin* 1.2 kb *5′HS4* insulator [19].

In order to investigate the function of the *D4Z4* subtelomeric repeat in the protection against CPE or TPE, we first asked whether a single repeat interferes *in cis* with the expression of an *eGFP* reporter gene using constructs stably integrated into the C33A human cells either randomly or at chromosome ends after telomeric fragmentation (Figure S1A). Chromosomal or Telomeric position effects (CPE or TPE respectively) are monitored by flow cytometry analysis (FACS) and manifest as variability from population to population in the percentage of cells expressing the *eGFP* reporter. In cells stably transfected with the T construct, telomere proximity reduces the percentage of eGFP positive cells compared to the pCMV vector that integrates randomly (Figure 1A, Figure S1B) both in polyclonal populations of transfected cells and in isolated clones as previously published [16],[17]. When inserted between a *de novo* formed telomere and *eGFP*, a single *D4Z4* has little effect on the expression of the reporter gene as indicated by the slight increase in eGFP positive cells in the population of cells carrying the T1X compared to cells containing the T construct (Figure 1A, Figure S1B). By contrast, *D4Z4* significantly increases the expression of the reporter construct inserted randomly in the genome (C1X *vs* pCMV, Figure 1A, *p*<0.0001 using a Student's *t*-test, Figure S1B). This effect is not attributable to an increased distance between the genomic environment at the site of integration and the reporter gene since progressive silencing is also observed after insertion of 3.5 kb of heterologous DNA (data not shown). Importantly, this potent effect of *D4Z4* on the expression of *eGFP* is not dependent upon the cell type since similar results were obtained in human rhabdomyosarcoma cells (TE671) and mouse myoblasts (C2C12) (Figure S1C). This first observation suggests that *D4Z4* acts either as an enhancer or an insulator by activating the *eGFP* reporter gene or protecting its expression from surrounding sequences. By cloning the *D4Z4* element upstream of the *eGFP* reporter or upstream of the *HyTK* resistance gene, we showed that *D4Z4* does not enhance the *eGFP* expression and concluded that it may act as an insulator (Figure S2).

Insulators are DNA sequences with two distinct properties. They can protect expression from the spreading of silent chromatin and CPE and they can uncouple promoter transcriptional activity from silencer and enhancer elements when inserted in between [18]. In order to discriminate between chromatin insulation and enhancer blocking activity for *D4Z4*, we performed enhancer blocking assays [19] by cloning *D4Z4* in sense (pNI-*D4Z4*-S) and antisense (pNI-*D4Z4*-AS) orientation into the pNI vector between the enhancer and the reporter (Figure 1B). *D4Z4* reduced the colony number in an orientation-independent way suggesting that it interferes with transcriptional enhancement. In order to distinguish insulation from repression, the right *β-globin* insulator (*5′HS4*) protecting from the influence of regulatory elements at the site of integration was replaced by *D4Z4* (p-E-*D4Z4*-S). In this configuration, the number of G418-resistant colonies is similar to the control indicating that *D4Z4* is not a repressor but showing that it protects the *γ-Neo* gene from the influence of repressive chromatin at the site of integration also in this context. Lastly, we tested the ability of *D4Z4* to enhance gene expression by removing the *5′ HS2* enhancer (E) in sense (p*D4Z4*-S) and antisense (p*D4Z4*-AS) constructs. In this assay, *D4Z4* does not activate *γ-Neo* expression. We conclude from these experiments that a single *D4Z4* is unable to activate or repress the expression of a reporter gene in a sense or antisense orientation, while it is able to block enhancer-promoter communications (Figure 1B). Overall, our findings indicate that *D4Z4* acts both as a transcriptional insulator (boundary element) protecting against the repressive influence of various chromosomal contexts and as an enhancer insulator interfering with enhancer-promoter communication. The *5′ HS4* insulator of the chicken *b-globin* locus [19] behaves similarly in our randomly integrated construct settings, albeit at a lower efficiency against CPE (Figure 1A).

**Figure 2:**
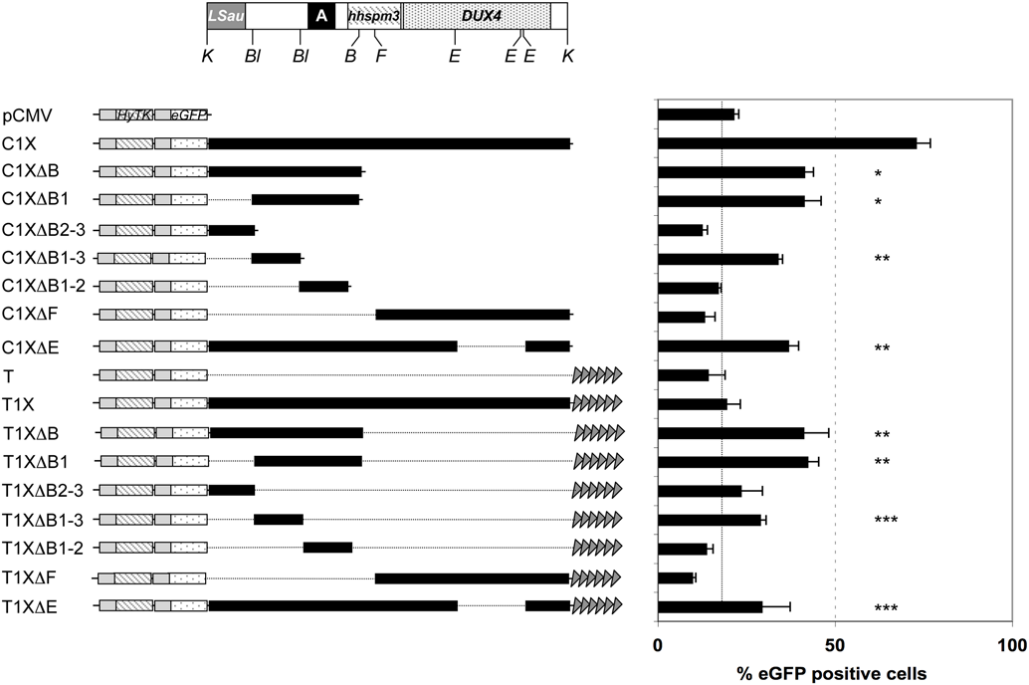
Mapping of the regulatory fragments within *D4Z4*. A. Schematic representation of the *D4Z4* element from position 1 to 3303 given relative to the two flanking *Kpn*I sites (K) (to scale). The different regions within *D4Z4* are indicated: *LSau* repeat (position 1–340), Region A (position 869–1071), *hhspm3* (position 1313–1780), *DUX4* ORF (position 1792–3063). The different restriction sites used for the cloning of *D4Z4* subfragments are indicated (B: *Bam*HI; Bl: *Blp*I; F: *Fse*I; E: *Ehe*I). B. Different fragments obtained after digestion of *D4Z4* were cloned downstream of the *eGFP* reporter (“C” constructs) or between the reporter gene and the telomeric seed (“T” constructs). Linearized plasmids were transfected into C33A cells and the percentage of eGFP positive cells was monitored by flow cytometry for an extended period of time. The histogram represents the mean value of the percentage of eGFP positive cells from day 18 to day 29 when *eGFP* expression reaches a plateau±S.D shown by error bars. Fragments DB2-3 (position 1 to 382), DB1-2 (position 814 to 1381) and DF (position 1549 to 3303) do not abrogate TPE or CPE while fragment DB1 (position 1 to 1381), DB1-3 (position 382 to 814) and DE (deleted of a distal 623 bp fragment from position 2269 to 2892) protect from CPE and TPE. Asterisks denote statistically significant values relative to control vectors (pCMV or T) (Student's t test). * *p*<0.001; **p<0.005; *** *p*<0.05.

Next, we mapped the portion of *D4Z4* responsible for this insulation activity by studying *eGFP* expression in constructs encompassing various truncated forms of *D4Z4*. Approximately half of the anti-silencing activity of *D4Z4* is present within a 432-bp region (position 382 to 814, C1XΔB1-3, Figure 2, *p*<0.0001 compared to pCMV using a Student's *t*-test,) called hereafter the proximal insulator. Interestingly, in contrast to the full-length *D4Z4* element, this proximal insulator also counteracts TPE suggesting an antagonistic effect between the proximal insulator and a distal silencer in a telomeric context (T1XΔB1-3, Figure 2). In agreement with this possibility, a construct containing the distal sequence (position 1549 to 3303) inserted either randomly (C1XDF) or terminally (T1XDF) is more repressed than the control *eGFP*. Moreover, a *D4Z4* sequence deleted of a distal 623 bp fragment (DE, deletion from position 2269 to 2892) recapitulates the insulator activity of the 1–1381 proximal fragment (Figure 2).

Thus, we conclude that much of the insulator activity against CPE and TPE is concentrated in the 432-bp proximal insulator while a silencer might be present in the distal portion of *D4Z4*.

### CTCF Binds to the Insulator Portion of *D4Z4*

Since in human cells, most insulators are bound by the multivalent CTCF protein [20],[21], we searched *in silico* for CTCF binding sites across the 3.3 kb *D4Z4* sequence using the consensus binding site at the chicken *β-globin* locus [22],[23]. Remarkably, the best matches were found within the proximal insulator, at position 468–481 and 476–489 (Figure S3A). Gel retardation assays were carried out to determine whether the sequence containing these two overlapping sites is capable of binding to CTCF (Figure S3B). The mobility was compared to the chicken *β-globin FII 5′HS4* site [22],[23] and we showed that both fragments produced a DNA-protein complex when incubated with nuclear extracts. These complexes can be disrupted by incubation with excess of unlabelled probes corresponding to the chicken *b-globin FII* or mouse *TAD1* [24] CTCF sites suggesting that CTCF specifically binds to the 5′ end of *D4Z4* (Figure S3B).

**Figure 3:**
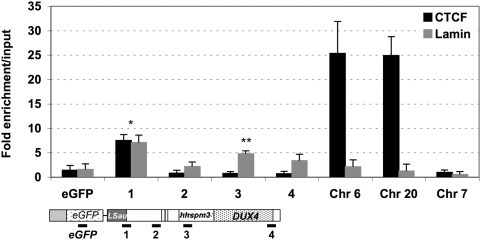
CTCF and A-type Lamins bind to *D4Z4 in vivo*. We searched *in silico* for CTCF binding sites across the 3.3 kb *D4Z4* sequence (Genbank accession number AF117653) using the consensus binding site at the chicken *β-globin locus* [22],[23]. Two sites were identified at the 5′ end of *D4Z4* and the binding was investigated by ChIP using antibodies to CTCF. We also investigated the involvement of A-type Lamins using specific antibodies. Enrichment of the immunoprecipitated DNA fraction with antibodies compared to input DNA was determined after real-time Q-PCR amplification (*y*-axis) for different primer pairs. Values were normalized to the Histone H4 internal standard. Each bar is the average of at least three independent experiments with the S.D. shown by *error bars*. “eGFP” amplifies the *eGFP* sequence. The position of the primers within *D4Z4* is indicated (sets 1–4). Using high-throughput analysis, numerous CTCF binding sites were recently identified and many of these sites also correspond to Cohesins enrichment [25]. We then asked if Cohesins/CTCF complex also contains A-Type Lamins and amplified DNA immunoprecipitated with Lamins A/C antibodies with primers corresponding to chromosome 6 (Chr 6). We observed a strong enrichment for CTCF but not Lamins at this site suggesting that CTCF/Cohesins and CTCF/Lamins bind distinct sites. A sequence on chromosome 20 (Chr 20) was reported as a site for CTCF only and does not bind A-type Lamins. Chr 7 primers are CTCF-negative control. Asterisks denote statistically significant values (** *p*<0.001; *p<0.005; Student's t test).

Furthermore, chromatin immunoprecipitation experiments (ChIP) reveal a nearly 8-fold enrichment for CTCF at this proximal insulator compared to an unrelated gene (Figure 3). Under these ChIP conditions, other known CTCF sites [25] on chromosomes 6 and 20 show a high level of enrichment, while no enrichment was observed for *D4Z4* regions located distally to the insulator, the adjacent *eGFP* gene or for an unrelated region on chromosome 7 (Figure 3). Importantly, this enrichment is lost in CTCF-depleted cells (Figure S3C, D).

### CTCF Is Necessary but not Sufficient for *D4Z4* Insulation

**Figure 4:**
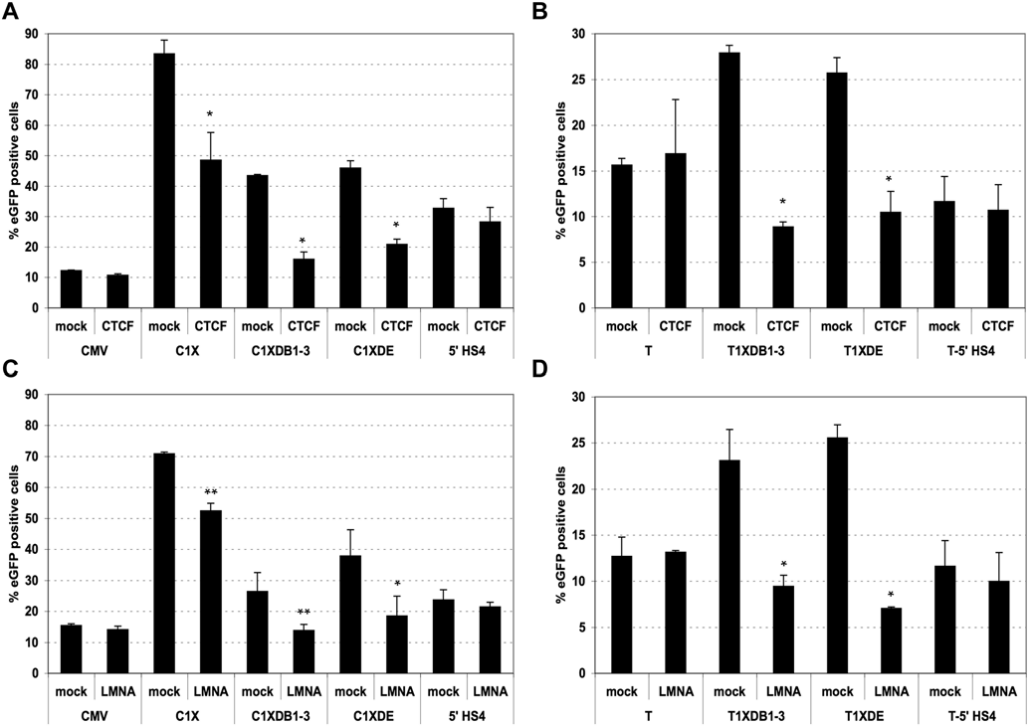
Depletion in CTCF or A-type Lamins abrogates *D4Z4* anti-silencing activity. The involvement of CTCF and A-type lamins in the insulating activity of *D4Z4* was studied by knocking-down their expression. A. Different populations of cells transfected with randomly integrated constructs were transfected with siRNA against *CTCF* (CTCF) or negative control siRNA (mock) and the level of eGFP was monitored by FACS. The mean value±S.D of three independent experiments are presented. B. Telomeric constructs harboring protection against TPE (T1XDB1-3, T1XDE) and control (T, T-5′ HS4) were transiently transfected with pools of siRNA against *CTCF*. The expression of the *eGFP* reporter gene was analyzed by FACS. C. D. The different constructs allowing the protection against CPE (C1X, C1XDB1-3, C1XDE, pCMV-5′ HS4) (panel C) or protection against TPE (T1XDB1-3, T1XDE) (panel D) were transiently transfected with siRNA against Lamins A/C and the expression of the *eGFP* reporter gene was measured by FACS. Asterisks denote statistically significant values relative to control siRNA (Student's t test). **p<0.01; *p<0.001.

Then, we tested the putative effect of CTCF on the insulator activity of *D4Z4* by transfecting the cells with different siRNAs that inhibit the expression of the *CTCF* gene (Figure S4A). The percentage of eGFP positive cells and intensity of fluorescence decrease in C1X cells subjected to a *CTCF* knock-down (KD) (Figure 4A) and in cells containing short fragments encompassing the proximal *D4Z4* insulator fragment either at the telomere (Figure 4B) or at random sites (Figure 4A, Figure S4C) suggesting that the knock-down of *CTCF* alters the anti-silencing properties of *D4Z4*. Of note, the effect of CTCF depletion observed with different siRNAs renders unlikely an off-target activity and does not significantly increases apoptosis in the time frame of the assay (Figure S4B). Moreover, it is also specific for CTCF since the reduced levels of another factor reported to bind to *D4Z4*, *YY1* [7], or proteins involved in the insulation properties of the chicken *5′HS4*, *USF1* and *2* [26], have no effect on *eGFP* expression (Figure S4E). Noteworthy, at the chicken *5′ HS4* insulator, CTCF is only involved in the enhancer blocking activity [27] and consistently, CTCF depletion does not modify the expression of the *eGFP* reporter protected by this insulator in our system suggesting that the mechanisms involved in the regulation of the *5′HS4* insulator and *D4Z4* are different.

Finally, there is no effect of *CTCF* KD in cells carrying a *D4Z4*-less construct (pCMV), showing that depletion of this protein does not alter the expression of our *eGFP* reporter in general but specifically impairs the insulator activity of *D4Z4* (Figure 4A).

Overall, these results show that CTCF is required for the insulation activity of *D4Z4*. Nevertheless, if CTCF is necessary for this property, it does not appear to be sufficient, since truncated forms of *D4Z4* containing the 5′ CTCF site only partially recapitulate the anti-silencing function of the whole repeat.

### A-Type Lamins Bind to *D4Z4* and Contribute to Its Insulation Activity

Since the localization of the 4q35 locus at the nuclear periphery is compromised in cells carrying a homozygous mutation of the *LMNA* gene [13], we hypothesized that A-type Lamins may contribute to *D4Z4* functions and we investigated this possibility by transfecting pools of siRNAs in different populations (Figure 4C,D). Depletion of A-type Lamins (Figure S4D) decreases the percentage of eGFP positive cells and intensity of fluorescence in cells carrying a randomly inserted *eGFP* reporter protected by the *D4Z4* insulator (C1X, C1XDB1-3; C1XDE cells, Figure 4C, Figure S4C). Interestingly, decreased insulation is also observed in cells containing the proximal *D4Z4* insulator element at chromosome ends (T1XDB1-3, T1XDE, Figure 4D) suggesting that A-type Lamins are necessary for the proper anti-silencing function of *D4Z4* and participate in the protection against TPE in the absence of the distal silencer element. However, *LMNA* knock-down does not change the level of eGFP in the absence of *D4Z4* (T construct) or in the presence of the *5′-HS4* insulator at chromosome ends (5′ HS4-T, Figure 4D). This effect is not merely the consequence of disruption of the nuclear periphery or depletion of components of the lamina since the knock-down of B-type Lamins (*LMNB*) or the Lamin A-associated protein, *BAF1* has not effect on *eGFP* expression (Figure S4E). Then, we investigated by ChIP whether A-type Lamins associate with *D4Z4*-tagged telomeres in our cellular model. We found that Lamins A/C are specifically enriched along the *D4Z4* repeat with a peak at the proximal insulator sequence where CTCF is bound (Figure 3) and concluded from this analysis that *D4Z4* interacts with A-type Lamins. These results show that Lamins A/C are involved in the anti-silencing activity of *D4Z4* and uncovers the involvement of both CTCF and A-type Lamins in the regulation of an insulator in human cells.

### The Multimerization of *D4Z4* Suppresses Protection against Silencing and CTCF Binding

**Figure 5:**
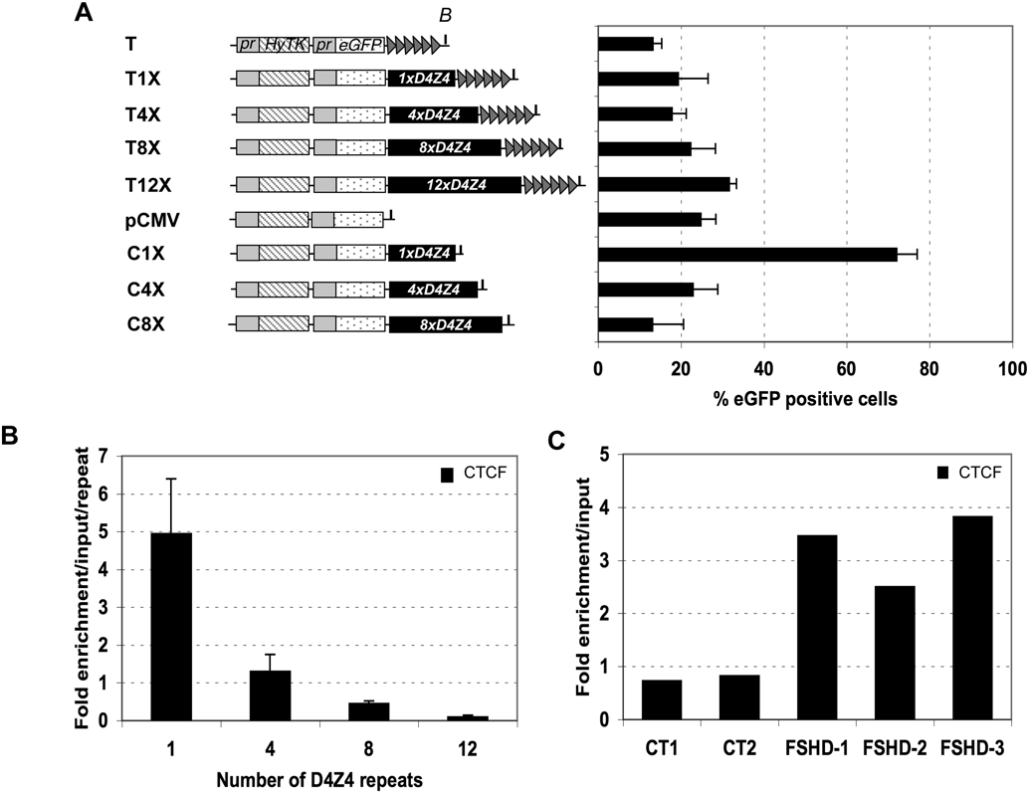
The multimerization of *D4Z4* abrogates CTCF binding and insulation activity. A. The expression of *eGFP* was measured by FACS on populations of cells transfected with telomeric constructs carrying 0, 1, 4, 8 or 12 copies of *D4Z4* downstream of the telomeric seed (T, T1X, T4X, T8X, T12X) or internal constructs (pCMV, C1X, C4X, C8X) containing respectively 0, 1, 4 or 8 copies of *D4Z4*. The integrity of each construct was verified in stable populations of cells after either random integration or telomeric fragmentation (Figure S1D). Histograms show the average percentage of eGFP positive cells from day 18 to day 29±S.D. shown by error bars, when *eGFP* expression reaches a plateau. In the different constructs containing *D4Z4* inserted at random sites (C4X, C8X), the level of eGFP is proportionally decreased when the number of repeats is increased suggesting that the repeated element loses its anti-CPE activity upon multimerization. On the opposite, eGFP level is slightly increased at telomeres (see main text). B. The binding of CTCF was investigated by ChIP on the different populations of cells carrying different number of *D4Z4* element downstream of the *eGFP* reporter gene. Input DNA and DNA fraction immunoprecipitated with antibodies to CTCF were amplified by a real-time Q-PCR method (*x*-axis) using primers encompassing the 5′ CTCF site. The *y*-axis shows the fold enrichment of CTCF in the bound fraction *versus* input chromatin. Each data point is the average of at least three independent experiments with the S.D. shown by *error bars*. C. ChIP analysis of CTCF binding in two different control (CT1 and CT2, >11 *D4Z4* repeats) and three different myoblasts from FSHD patients (FSHD1, 5 repeats; FSHD 2, 6 repeats; FSHD 3, 7 repeats).

Since *D4Z4* is repeated in tandem at several chromosomal *loci*, including the 4q subtelomeres where it is linked to FSHD, we explored whether the multimerization of *D4Z4* alters its properties. At telomeres, adding up to 12 copies of *D4Z4* slightly weakens telomeric silencing suggesting that a large *D4Z4* array may act as a fuzzy boundary shielding from the repressive effect of telomeric chromatin when *D4Z4* directly abuts the telomere (Figure 5A). However, this situation might not directly reflect the natural genomic context since the distance between *D4Z4* and the telomere is estimated to be around 25–50 kb and other subtelomeric sequences might also exert an effect on the *D4Z4* arrays.

We showed that adding up to 8 *D4Z4* elements progressively abolishes the insulation activity in randomly integrated constructs suggesting that the repeated element looses its anti-silencing activity upon multimerization (Figure 5A). Consistent with this hypothesis, loss of anti-silencing correlates with the loss of CTCF binding (Figure 5B) and a slight increase in the trimethylation of lysine 9 residues on histone H3 tails, a mark of silenced chromatin (Figure S5). Impressively, the gain in CTCF binding was also observed in myoblasts from FSHD patients compared to controls suggesting that the binding of CTCF to the *D4Z4* repeats is a molecular marker of FSHD muscle (Figure 5C). We propose that reduction of the *D4Z4* array in FSHD patients allows the binding of CTCF and provokes changes in the biological function of *D4Z4* that switches from a repressor to an insulator protecting the expression of the FSHD gene(s).

## Discussion

**Figure 6:**
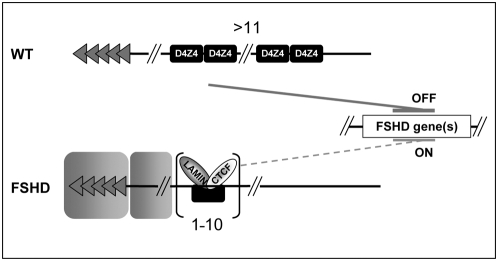
Model explaining the role of the *D4Z4* insulator and its implication in the epigenetic alteration of FSHD. In normal cells, the multimerization of *D4Z4* compromises CTCF binding and the boundary activity is counteracted (upper panel). In this conformation, the *D4Z4* array might repress gene expression either at the 4q35 locus or at a long distance from the array. In patients, *D4Z4* acts as an insulator that protects the expression of different *loci* from repressive structures such as the 4q terminus or other subtelomeric surrounding sequences. This boundary activity depends upon CTCF and Lamins A/C (lower panel). The exclusion of CTCF from multiple repeats and the presence of a silencer element within *D4Z4* [7] might suggest that the *D4Z4* array behaves as a silencer. However, the presence of up to 12 copies of the repeat does not repress the expression of the neighboring *eGFP* reporter in our experimental settings where the *D4Z4* array directly flanks the telomere and argues against the hypothesis that multiple *D4Z4* repress in *cis* the expression of genes. An alternative explanation is that multiple *D4Z4* cooperates with other elements of the 4q region to form a silencer, as suggested by the link between *D4Z4* array contraction and a particular allele of 4q35 in patients [37].

By analyzing the behavior of the *D4Z4* subtelomeric element in various chromosomal settings, we demonstrated that this repeat behaves as a CTCF and A-type Lamins-dependent transcriptional insulator. These features are specific for *D4Z4* since they are not shared by the CTCF-dependent *5′HS4 b-globin* insulator where CTCF only shields against enhancer-promoter communication [27]. As a single repeat, *D4Z4* binds to CTCF and A-type Lamins, behaves as a transcriptional insulator, preventing both the communication between a *cis*-regulatory element and a promoter (enhancer blocking activity) and protecting against chromosomal position effect (anti-silencing activity) (Figure 6). Upon multimerization of *D4Z4*, CTCF binding is impaired. Thus, the experiments presented here reveal a novel mode of chromatin regulation controlled by the number of *D4Z4* repeats. Furthermore, our data uncover a novel property for A-type Lamins in the protection against CPE in human cells as observed in *Drosophila* [28],[29],[30] and suggested by the previous co-purification of A-type Lamins and CTCF in HeLa cells [31].

The association between the CTCF-dependent *5′ HS4* insulator and the nucleolus [31] and recent data showing that Cohesins [25],[32] or Emerin and B-type Lamins [33] can colocalize with CTCF throughout the genome, suggest that this protein interacts with different key components of the nuclear architecture to mediate transcriptional insulation and organization of human chromosomes. In agreement with the possible existence of different classes of CTCF-dependent insulators, we have been unable to detect a significant enrichment of Lamins A/C at the CTCF sites at chromosomes 6 and 20 (Figure 3) and the KD of nucleolin or *SCC1* [25] has no effect on the antisilencing activity of *D4Z4* (Figure S6). Together with a recent publication on an unrelated macrosatellite repeat on the X chromosome [34], our work further extends the notion that CTCF is implicated in the functional organization of the genome by showing that it interacts also with repeated elements and suggests a genome-wide role in the formation of chromatin boundaries at the transition between transcribed regions and silenced chromatin through the association with different specialized complexes and may thereby direct the corresponding chromosome segment to specialized subnuclear compartments.

Importantly, we showed that CTCF is specifically recruited to the 4q35 region of FSHD patients. Similar observations were made by G. Fillipova & colleagues (personnal communication). The relationship between a reduced number of *D4Z4* sequences and a gain-of-function of a CTCF-dependent insulation activity suggests an alternative mechanism for FSHD physiopathology based on a switch of activity from a repressor to an insulator element (Figure 6). Indeed, most FSHD patients have less than 11 copies of *D4Z4* at 4q35 and the severity of the disease negatively correlates with the number of residual repeats [35],[36]. The shortening of the array would both eliminate the silencer properties of *D4Z4* [7] and unmask an insulator function that may protect the FSHD genes from silencing emanating either from the 4q terminus or the β-satellite-rich region on the 4qA allele that was reported to co-segregate with the disease [37],[38]. Noticeably, in the human genome, β-satellite elements are often found in the vicinity of *D4Z4* repeats [39] raising the possibility for a role of the *D4Z4* insulator as a barrier between euchromatin and heterochromatin-like sequences. Since the *D4Z4* array is hypermethylated when present in high copy number [40] and since CTCF binding can be compromised by DNA methylation [22],[41] or block the spreading of this DNA modification [21], a likely hypothesis is that CTCF binding modulates the biological function of *D4Z4* in cooperation with changes in the pattern of DNA methylation. However, in agreement with previous observation [42],[43], the silencing activity observed upon multimerization of *D4Z4* does not seem to be associated with a massive heterochromatinization of the array of repeats (Figure S5). Therefore, we propose a model in which the loss of CTCF binding changes the spatial configuration of the region rather than the condensation of the chromatin of the locus.

Our results also implicate CTCF and A-type Lamins as important players in FSHD. In agreement with this notion, patients with FSHD display some clinical and transcriptional resemblances to Emery-Dreifuss, a muscular dystrophy linked to mutation in the *LMNA* gene [44], suggesting that the affinity of *D4Z4* for A-type lamins might contribute to the epigenetic regulation of the 4q35 locus by providing the proper subnuclear environment for the regulation of the gene(s) causing the dystrophy. Together with the matrix attachment sites at the 4q35 *locus* [15], the subnuclear localization of the 4q35 locus at the edge of the nucleus [13],[14] and the positioning activity of *D4Z4* at the nuclear periphery (Ottaviani et al., submitted), the association of *D4Z4* to these two proteins might create functional domains involved in insulation mechanisms. Although one cannot exclude that soluble lamins A/C are bound to *D4Z4*, one can speculate that depending on the position of the locus at the nuclear periphery, the lamina may either provide a high concentration of regulatory factors or favor looping between distant sequences. With respect to FSHD, the corollary of this hypothesis is that the pathogenic 4q35 allele carrying a shortened *D4Z4* array might be repositioned along the inner nuclear envelope from a repressive to a permissive compartment modulating thereby the microenvironment of the genes causing the disease.

Thus, beyond the importance of a better characterization of *D4Z4* for its relevance to the peculiar Facio-Scapulo-Humeral dystrophy, this work reveals the existence of a human insulator element that depends on both CTCF and A-type Lamins. In addition, this work suggests that the mosaic nature of human subtelomeres might directly influence the higher-order organization of the corresponding chromosome end. This may serve as a paradigm for our understanding of numerous pathologies linked to subtelomeres such as idiopathic mental retardation.

## Materials and Methods

### Ethics Statement

This study was conducted according to the principles expressed in the Declaration of Helsinky. The study was approved by the Institutional Review Board. All patients provided written informed content for the collection of samples and subsequent analysis.

### Cellular Models

The pCMV and pCMVTelo plasmids are described in Koering *et al* [17]. Experimental details and characterization of the cell lines are given in Text S1.

### RNA Interference

We used pre-annealed small interfering RNAs (siRNAs): siGENOME SMARTpool reagent, human *CTCF* (M-020165-01); human *LMNA* (NM_170707) (Dharmacon), Silencer™ Negative Control #1 siRNA (Ambion). Transfections were performed with DharmaFECT 1™ (Dharmacon) with 200 pmoles siRNA for 2×10^5^cells. Efficient knock-down was determined by quantitative RT-PCR or Western blot. See Text S1.

### Chromatin Immunoprecipitation

*In vivo* protein-DNA cross-linking was carried out as described [24]. Nucleoprotein complexes were sonicated to reduce DNA fragments to 400–600 bp using a Bioruptor sonifier (Diagenode). Immunoprecipitation was performed with a rabbit polyclonal anti-CTCF (Upstate Biotechnologies, ref 07-729) or a goat polyclonal anti-Lamins A/C (Santa Cruz, ref SC6215, [45],[46]). After immunoprecipitation, DNA samples were quantified using the NanoDrop ND-1000 spectrophotometer (NanoDrop technologies) and enrichment of the immunoprecipitated fraction was quantified by Real Time Q-PCR (Text S1).

## Supporting Information

Figure S1 Contraction of the *D4Z4* array unmasks a boundary activity. A. Description of the seeding constructs and procedure. Telomere seeding is based on the non-targeted introduction of cloned telomeres into mammalian cells. The constructs carry a hygromycin resistance gene fused to the herpes simplex virus type 1 thymidine kinase suicide gene (*HyTK*), an *eGFP* reporter gene, both driven by CMV promoters. We inserted *D4Z4* between the reporter and the telomere in order to investigate the effect of *D4Z4* on gene expression. The transfection of constructs linearized downstream of a 1.2 kb (TTAGGG)n seed of human telomeric repeats (*Bst*XI site, B) allows *de novo* telomere formation at the integration site while constructs lacking these repeats integrate randomly in the host genome. Conditions of transfection of the C33A cell line were optimized in order to have a single integration of the transgene per cell. Successful *de novo* formation of *eGFP*-tagged telomeres and single integration was confirmed in the polyclonal population of transfected cells and in a set of clones by fluorescence *in situ* hybridization (FISH) on metaphase spreads (as illustrated in photographs 1, 2 for telomeric insertion and in photographs 3, 4 for internal integration) and by detection of a diffuse hybridization signal in Southern blot (data not shown). In agreement with previous data, the rate of *de novo* telomere formation in stably transfected cells is very high in the C33A cells reaching 80–90% of the hygromycin resistant cells for the T and T1X constructs. We also confirmed by Multiplex FISH analysis that in the presence of *D4Z4*, the constructs do not integrate at preferential sites (Ottaviani et al., Submitted). Three days after transfection, Hygromycin B was added to the medium. Then, cells were grown for an extended time in selective medium. The percentage of eGFP-positive cells and the average level of eGFP were monitored by Flow Cytometry (FACS) every 3 days for up to 90 days. B. Kinetics of the expression of *eGFP*. After 10–12 days, the percentage of eGFP-positive cells decreases in cells containing the T construct that plateaus at 10–20%. A low *eGFP* expression is also observed in cells carrying a single *D4Z4* element at a subtelomeric position (T1X). On the opposite, the level of eGFP is high in C1X cells and remains constant throughout the course of the assays. C. The pCMV and C1X constructs were transfected into C2C12 mouse myoblasts or a human rhabdomyosarcoma cell line (TE671) and the level of eGFP was monitored by FACS for up to 30 days. As previously described for C33A cells, *D4Z4* protects the expression of the *eGFP* from CPE in the different cell types tested indicating that insulation mediated by *D4Z4* is not dependent upon the cell type. D. The integrity of each construct was verified by Quantitative PCR from genomic DNA of hygromycin resistant cells using different set of primers. The Ct values obtained for each construct were normalized to the H4 promoter as an internal control and compared to the values obtained for the T construct containing only the resistance gene, eGFP reporter and the telomere seed. Untransfected C33A cells were used as negative control (data not shown). For each primer set, the average fold-increase from 3 independent cell populations (±S.D. shown by error bars) is indicated in representative populations of cells. The *eGFP* sequence and the 3′ end of the construct can be detected in the different populations while the number of *D4Z4* increases in cells transfected with vectors containing multiple copies of the repeats.
(0.7 MB TIF)

Figure S2 *D4Z4* does not enhance *eGFP* expression. In order to test the role of *D4Z4* in the control of gene expression, the repeat was cloned upstream of the pCMV promoter driving the *eGFP* reporter (1XC construct) or upstream of the pCMV promoter driving the *HyTK* resistance gene (X1C construct) and compared to the pCMV control vector or the C1X construct. When present upstream of the *eGFP* reporter or the HyTK gene, *D4Z4* does not enhance the expression of the reporter indicating that *D4Z4* does not act as a transcriptional enhancer in these situations.
(8.3 MB TIF)

Figure S3 CTCF binds to *D4Z4 in vitro*. To determine whether the candidate CTCF binding sequences (A) are capable of binding to CTCF, gel retardation assays were carried out (B). The mobility was compared to the chicken *b globin FII 5′HS4* site. The *FII* (lane 1) and *D4Z4* CTCF site (lane 5) can be supershifted by incubation with a CTCF antibody (star). We also used unlabelled oligonucleotides corresponding to known CTCF binding sites for competition assays. C33A nuclear extracts were incubated either with labeled *FII* (lanes 1–4) or 468-S labeled oligonucleotides (lanes 5–8) and molar excess of *FII* (lanes 3, 7) or TAD1 site at the mouse *TCRα*-*Dad1 locus* [24] (lanes 4, 8). Molar excess of unlabeled *FII* or *TAD1* can displace the binding of CTCF from the labeled *D4Z4* sequence whereas mutant versions of *FII* cannot (data not shown) suggesting that the sites at position 468–481 and 476–489 of *D4Z4* bind CTCF. C. Different primer sets spanning the construct were used to amplify input DNA and DNA fraction immunoprecipitated with antibodies to CTCF by a real-time Q-PCR method. The *y*-axis shows the fold enrichment of CTCF in the bound fraction *versus* input chromatin. Each data point indicates the average of at least three independent experiments with the S.D. shown by *error bars*. A significant enrichment of more than 7-fold was observed with primers encompassing the putative CTCF binding site showing that CTCF interacts with the *D4Z4* repeat *in vivo* (black bars). This enrichment is lost when chromatin immunoprecipitation is performed on cells transfected with siRNA against *CTCF* (grey bars). D. Schematic representation of *D4Z4* with the position of the primers used for ChIP quantification.
(8.3 MB TIF)

Figure S4 Validation of CTCF and A-type Lamins knock-down. A. C1X and pCMV cell populations were transfected with pools of siRNA against *CTCF* (pool CTCF), 3 different siRNAs (CTCF 1, 2, 3) or negative control siRNA (sineg) and quantification of *CTCF* and *eGFP* mRNA was performed by reverse transcription followed by quantitative PCR amplification. The values were normalized to the b-Actin standard. The percentage of *CTCF* or *eGFP* mRNA for cells treated with *CTCF* siRNA *vs* control cells is indicated. B. CTCF is a versatile protein that regulates numerous pathways in human cells. In order to verify that the KD of *CTCF* does not affect cell viability and subsequently, eGFP level, cell populations were incubated with BrdU 7 days after transfection and cell cycle was analyzed by flow cytometry. No significant difference could be observed in cells transfected with negative control siRNA (mock) compared to *CTCF* siRNA (CTCF). C. A population of cells stably transfected with the CDE construct were transiently transfected with negative control siRNA (mock) or siRNA against *CTCF* or A-type Lamins (*LMNA*). The percentages of eGFP positive cells were determined by FACs three days after transfection. The leftward shift peak in cells transfected with siRNA to *CTCF* or *LMNA* indicates that the intensity of the eGFP is decreased in the pool of eGFP positive cells compared to control cells. D. Different cell populations were transfected with pools of siRNA against products of the *LMNA* gene. Depletion in A and C type lamins was controlled by western blot on whole cell extracts 4 days (1T) or 7 days (1T+3 days) after a first transient transfection or 4 days after a second transfection (2T) and compared to the level of both proteins in mock-treated cells. A goat polyclonal antibody was used for western blot and ChIP experiments. The total amount of protein in each extract was compared by using an anti-actin antibody E. The specificity of the CTCF and Lamins effects on the activity of *D4Z4* was compared to the effect of YY1 that was previously reported to bind to *D4Z4* [7], USF1 and 2 that participate in the insulator activity of the chicken *5′HS4* insulator [26] and components of the nuclear Lamina, Lamin B or BAF1. Therefore, T1XDE cells were transiently transfected with pools of siRNA against the different genes or negative control siRNA (mock). The % of eGFP positive cells was determined by FACS 3 to 7 days after transfection and we did not observe a significant decrease in eGFP level in the different populations of transfected cells. Similar results were observed for the other constructs harboring insulator activity.
(0.4 MB TIF)

Figure S5 Loss of CTCF binding only slightly increases the trimethylation of H3 K9 residues. Above the threshold of 11 copies, the *D4Z4* array is methylated at the DNA level suggesting that long stretches of *D4Z4* become more condensed. CTCF might be important in the control of the chromatin structure and we wanted to test if the loss of CTCF binding that we observed upon *D4Z4* multimerization is accompanied by an increase in the trimethylation lysine 9 residues on histone H3 tails. Therefore, ChIP was performed with anti-Me_3_-H3K9 in cells stably transfected with different *D4Z4* vectors. Values were normalized to the histone H4 promoter as a standard and enrichments of the immunoprecipitated DNA compared to input DNA are presented (*y-axis*).
(1.1 MB TIF)

Figure S6 Cohesins do not participate in the *D4Z4* insulator activity. Recently, high throughput techniques allowed the identification of numerous binding sites for Cohesins [25],[32],[51] throughout the human genome. Interestingly, many of these sites also correspond to CTCF sites suggesting that the two proteins might be involved in insulation activity. In order to see whether Cohesins also contribute to *D4Z4* activity we transfected the T1XΔE cells with siRNA against *SCC1* [25] and measured the expression of *eGFP* 3 to 7 days after transfection. We did not observe a significant difference after transfection of these siRNA suggesting that Cohesins do not contribute to the activity of *D4Z4*.
(1.1 MB TIF)

Text S1 Supplementary information.
(0.09 MB DOC)
