# Supplementary figures and images for: The D4Z4 Macrosatellite Repeat Acts as a CTCF and A-Type Lamins-Dependent Insulator in Facio-Scapulo-Humeral Dystrophy
Source: PLoS Genet. 2009 Feb 27;5(2):e1000394. doi: 10.1371/journal.pgen.1000394 (PMC2639723; doi:10.1371/journal.pgen.1000394)

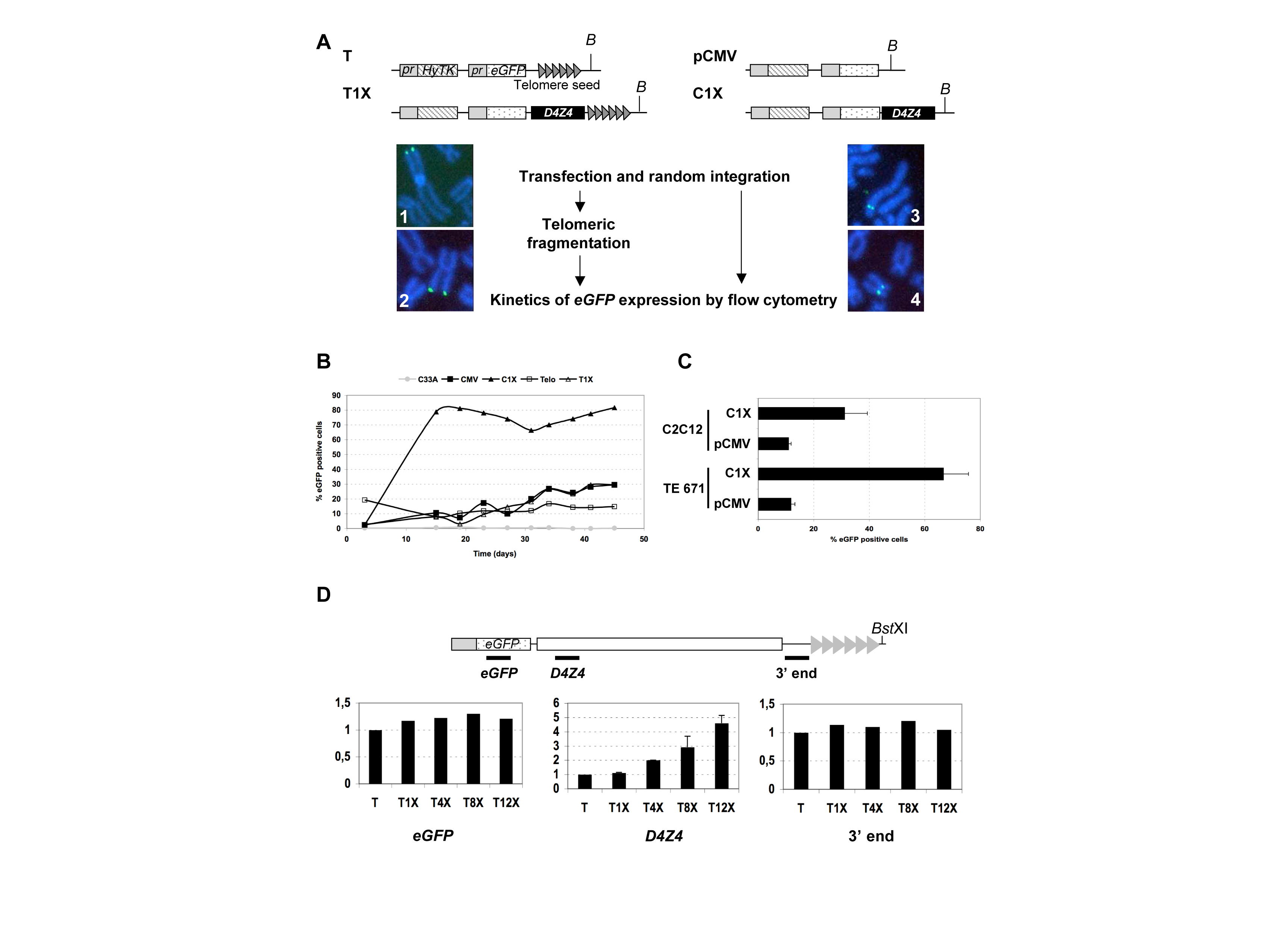

Supplement: Figure S1 — Contraction of the D4Z4 array unmasks a boundary activity. A. Description of the seeding constructs and procedure. Telomere seeding is based on the non-targeted introduction of cloned telomeres into mammalian cells. The constructs carry a hygromycin resistance gene fused to the herpes simplex virus type 1 thymidine kinase suicide gene (HyTK), an eGFP reporter gene, both driven by CMV promoters. We inserted D4Z4 between the reporter and the telomere in order to investigate the effect of D4Z4 on gene expression. The transfection of constructs linearized downstream of a 1.2 kb (TTAGGG)n seed of human telomeric repeats (BstXI site, B) allows de novo telomere formation at the integration site while constructs lacking these repeats integrate randomly in the host genome. Conditions of transfection of the C33A cell line were optimized in order to have a single integration of the transgene per cell. Successful de novo formation of eGFP-tagged telomeres and single integration was confirmed in the polyclonal population of transfected cells and in a set of clones by fluorescence in situ hybridization (FISH) on metaphase spreads (as illustrated in photographs 1, 2 for telomeric insertion and in photographs 3, 4 for internal integration) and by detection of a diffuse hybridization signal in Southern blot (data not shown). In agreement with previous data, the rate of de novo telomere formation in stably transfected cells is very high in the C33A cells reaching 80–90% of the hygromycin resistant cells for the T and T1X constructs. We also confirmed by Multiplex FISH analysis that in the presence of D4Z4, the constructs do not integrate at preferential sites (Ottaviani et al., Submitted). Three days after transfection, Hygromycin B was added to the medium. Then, cells were grown for an extended time in selective medium. The percentage of eGFP-positive cells and the average level of eGFP were monitored by Flow Cytometry (FACS) every 3 days for up to 90 days. B. Kinetics of the expres [file pgen.1000394.s001.tif]

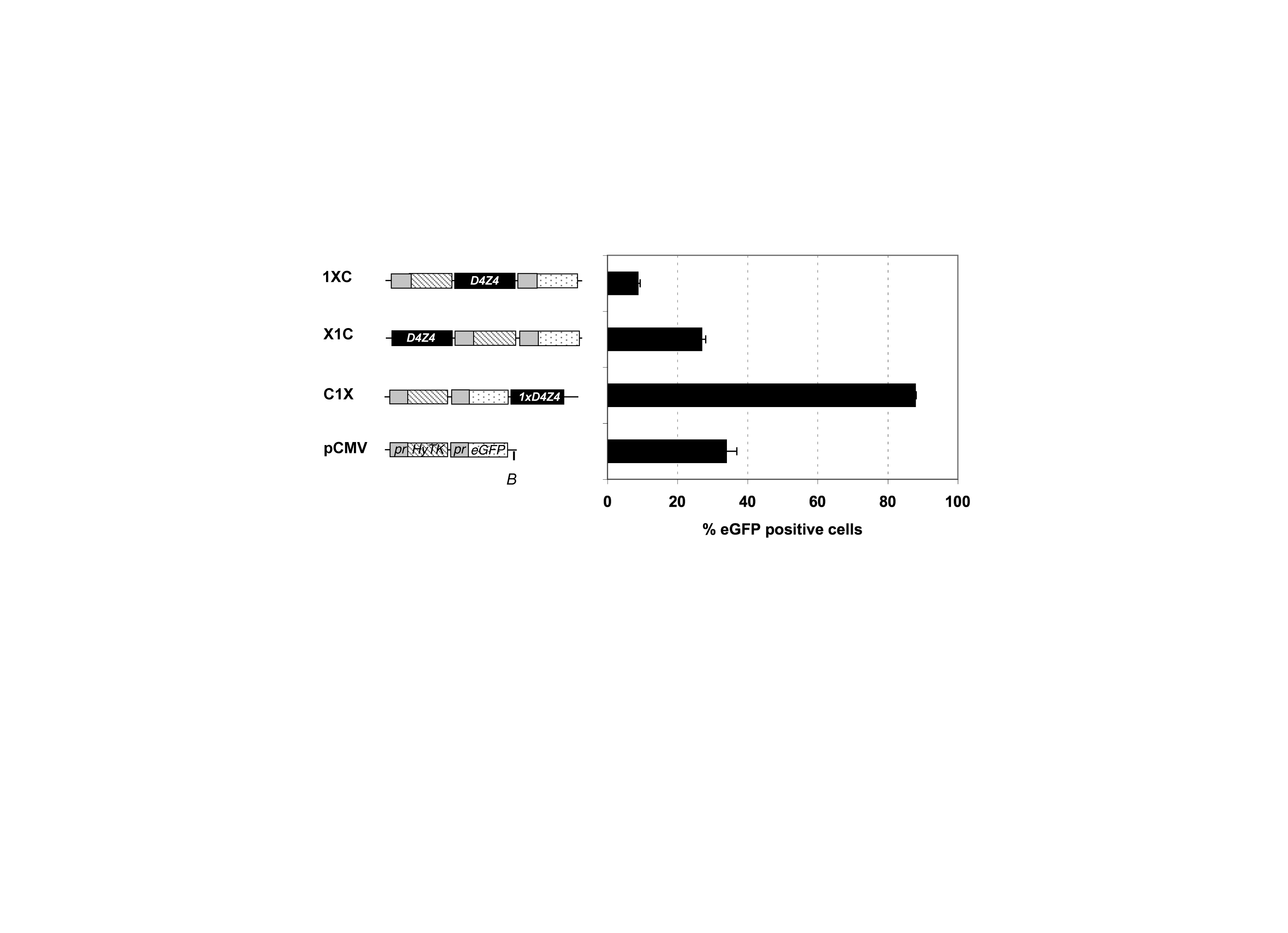

Supplement: Figure S2 — D4Z4 does not enhance eGFP expression. In order to test the role of D4Z4 in the control of gene expression, the repeat was cloned upstream of the pCMV promoter driving the eGFP reporter (1XC construct) or upstream of the pCMV promoter driving the HyTK resistance gene (X1C construct) and compared to the pCMV control vector or the C1X construct. When present upstream of the eGFP reporter or the HyTK gene, D4Z4 does not enhance the expression of the reporter indicating that D4Z4 does not act as a transcriptional enhancer in these situations. (8.3 MB TIF) [file pgen.1000394.s002.tif]

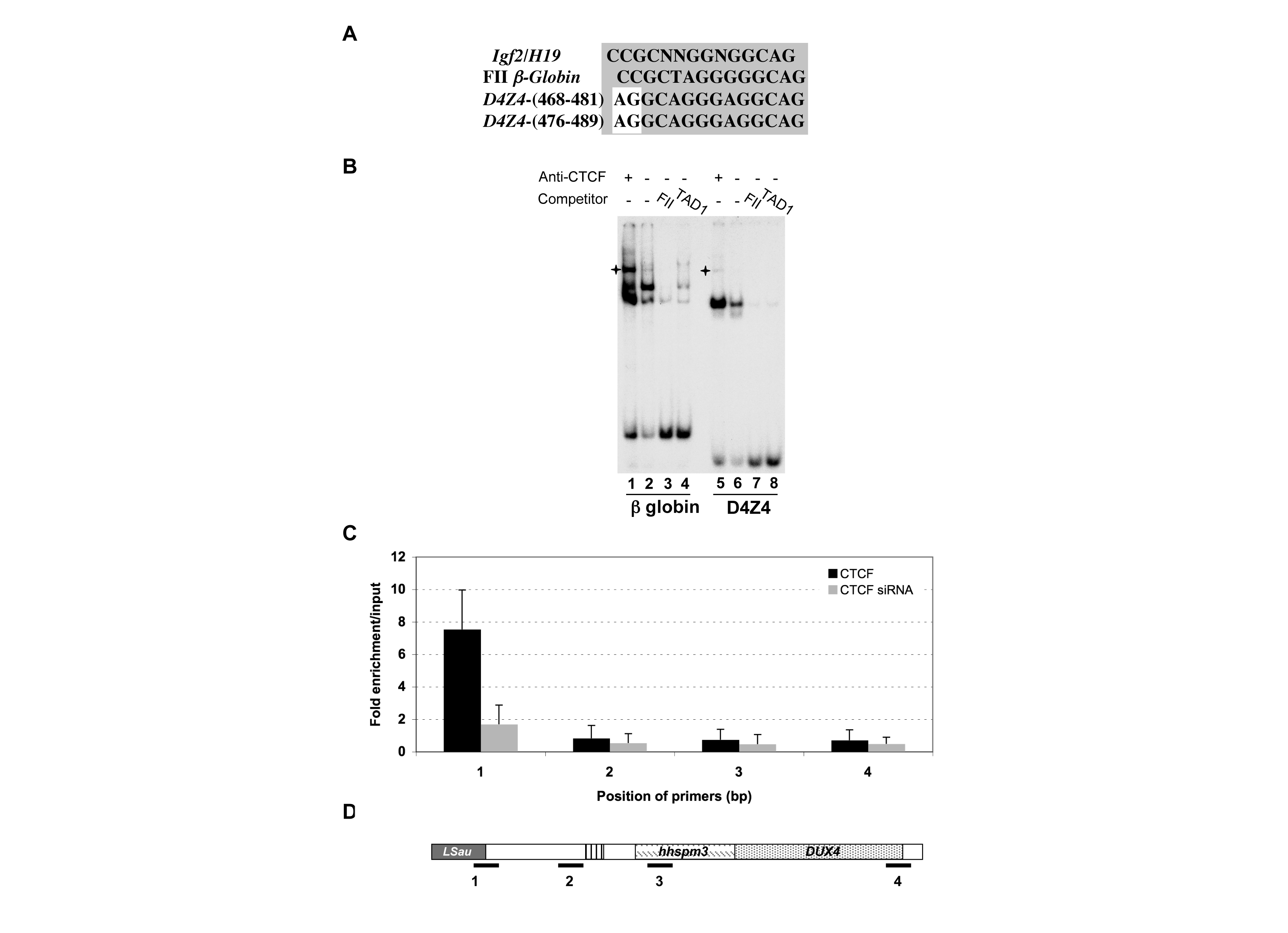

Supplement: Figure S3 — CTCF binds to D4Z4 in vitro. To determine whether the candidate CTCF binding sequences (A) are capable of binding to CTCF, gel retardation assays were carried out (B). The mobility was compared to the chicken b globin FII 5′HS4 site. The FII (lane 1) and D4Z4 CTCF site (lane 5) can be supershifted by incubation with a CTCF antibody (star). We also used unlabelled oligonucleotides corresponding to known CTCF binding sites for competition assays. C33A nuclear extracts were incubated either with labeled FII (lanes 1–4) or 468-S labeled oligonucleotides (lanes 5–8) and molar excess of FII (lanes 3, 7) or TAD1 site at the mouse TCRα-Dad1 locus [24] (lanes 4, 8). Molar excess of unlabeled FII or TAD1 can displace the binding of CTCF from the labeled D4Z4 sequence whereas mutant versions of FII cannot (data not shown) suggesting that the sites at position 468–481 and 476–489 of D4Z4 bind CTCF. C. Different primer sets spanning the construct were used to amplify input DNA and DNA fraction immunoprecipitated with antibodies to CTCF by a real-time Q-PCR method. The y-axis shows the fold enrichment of CTCF in the bound fraction versus input chromatin. Each data point indicates the average of at least three independent experiments with the S.D. shown by error bars. A significant enrichment of more than 7-fold was observed with primers encompassing the putative CTCF binding site showing that CTCF interacts with the D4Z4 repeat in vivo (black bars). This enrichment is lost when chromatin immunoprecipitation is performed on cells transfected with siRNA against CTCF (grey bars). D. Schematic representation of D4Z4 with the position of the primers used for ChIP quantification. (8.3 MB TIF) [file pgen.1000394.s003.tif]

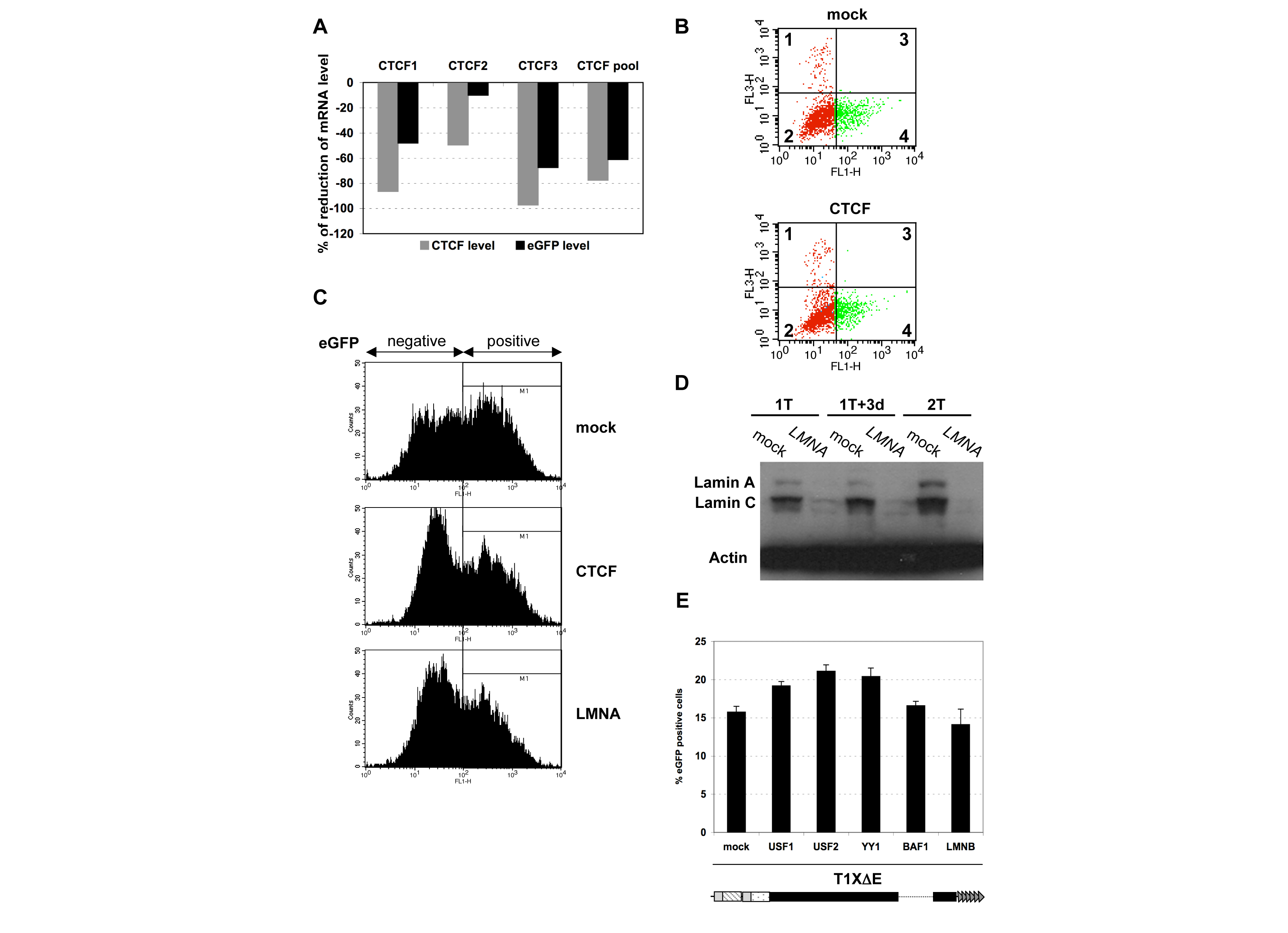

Supplement: Figure S4 — Validation of CTCF and A-type Lamins knock-down. A. C1X and pCMV cell populations were transfected with pools of siRNA against CTCF (pool CTCF), 3 different siRNAs (CTCF 1, 2, 3) or negative control siRNA (sineg) and quantification of CTCF and eGFP mRNA was performed by reverse transcription followed by quantitative PCR amplification. The values were normalized to the b-Actin standard. The percentage of CTCF or eGFP mRNA for cells treated with CTCF siRNA vs control cells is indicated. B. CTCF is a versatile protein that regulates numerous pathways in human cells. In order to verify that the KD of CTCF does not affect cell viability and subsequently, eGFP level, cell populations were incubated with BrdU 7 days after transfection and cell cycle was analyzed by flow cytometry. No significant difference could be observed in cells transfected with negative control siRNA (mock) compared to CTCF siRNA (CTCF). C. A population of cells stably transfected with the CDE construct were transiently transfected with negative control siRNA (mock) or siRNA against CTCF or A-type Lamins (LMNA). The percentages of eGFP positive cells were determined by FACs three days after transfection. The leftward shift peak in cells transfected with siRNA to CTCF or LMNA indicates that the intensity of the eGFP is decreased in the pool of eGFP positive cells compared to control cells. D. Different cell populations were transfected with pools of siRNA against products of the LMNA gene. Depletion in A and C type lamins was controlled by western blot on whole cell extracts 4 days (1T) or 7 days (1T+3 days) after a first transient transfection or 4 days after a second transfection (2T) and compared to the level of both proteins in mock-treated cells. A goat polyclonal antibody was used for western blot and ChIP experiments. The total amount of protein in each extract was compared by using an anti-actin antibody E. The specificity of the CTCF and Lamins effects on the activity of D4Z4 was compared to t [file pgen.1000394.s004.tif]

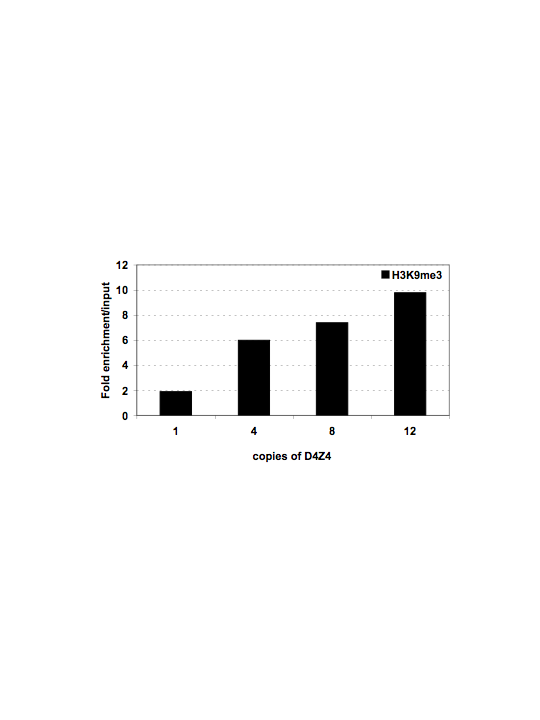

Supplement: Figure S5 — Loss of CTCF binding only slightly increases the trimethylation of H3 K9 residues. Above the threshold of 11 copies, the D4Z4 array is methylated at the DNA level suggesting that long stretches of D4Z4 become more condensed. CTCF might be important in the control of the chromatin structure and we wanted to test if the loss of CTCF binding that we observed upon D4Z4 multimerization is accompanied by an increase in the trimethylation lysine 9 residues on histone H3 tails. Therefore, ChIP was performed with anti-Me3-H3K9 in cells stably transfected with different D4Z4 vectors. Values were normalized to the histone H4 promoter as a standard and enrichments of the immunoprecipitated DNA compared to input DNA are presented (y-axis). (1.1 MB TIF) [file pgen.1000394.s005.tif]

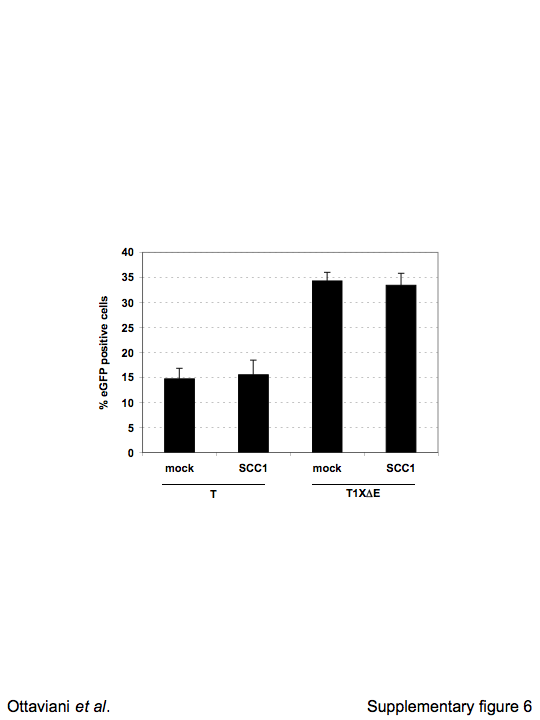

Supplement: Figure S6 — Cohesins do not participate in the D4Z4 insulator activity. Recently, high throughput techniques allowed the identification of numerous binding sites for Cohesins [25],[32],[51] throughout the human genome. Interestingly, many of these sites also correspond to CTCF sites suggesting that the two proteins might be involved in insulation activity. In order to see whether Cohesins also contribute to D4Z4 activity we transfected the T1XΔE cells with siRNA against SCC1 [25] and measured the expression of eGFP 3 to 7 days after transfection. We did not observe a significant difference after transfection of these siRNA suggesting that Cohesins do not contribute to the activity of D4Z4. (1.1 MB TIF) [file pgen.1000394.s006.tif]
