# Supplementary material for: The D4Z4 Macrosatellite Repeat Acts as a CTCF and A-Type Lamins-Dependent Insulator in Facio-Scapulo-Humeral Dystrophy
Source: PLoS Genet. 2009 Feb 27;5(2):e1000394. doi: 10.1371/journal.pgen.1000394 (PMC2639723; doi:10.1371/journal.pgen.1000394)
Supplement: Text S1 — Supplementary information. (0.09 MB DOC) [file pgen.1000394.s007.doc]

**Supplementary information.**

**Materials & Methods**

**Cell culture.** The epithelial cervix adenocarcinoma C33-A, rhabdomyosarcoma TE671 human cell lines and mouse C2C12 myoblasts were maintained in Dulbecco’s Modified Eagle’s Medium (D-MEM) with L-alanyl-L-glutamine, D-glucose and sodium pyruvate (Invitrogen). The human erythroleukemia cell line K562 was maintained in Improved Minimal Essential Medium (IMEM). Media were supplemented with 10% FBS (Invitrogen) and 1% Penicillin-Streptomycin, 10000 units/ml (Invitrogen). All cell lines were grown at 37°C, 5% CO_2_ , in a humidified atmosphere.

**Myoblast selection.** Four FSHD patients carrying respectively 5 (affected sub scapular muscle), 6 (unaffected quadriceps) and 7 (unaffected quadriceps) repeats and two controls were included after giving their informed consent. Myoblasts were prepared as previously described [48] .

**Constructs, transfections and flow cytometry.** The pCMV and pCMVTelo (named thereafter “T”) plasmids are described in Koering *et al* [17]. The *D4Z4* was cloned at the *Fse*I site of the two basic vectors. Additional *D4Z4* fragments were subsequently inserted between the first *D4Z4* and the telomere. For constructs containing 8 or 12 copies of *D4Z4*, the reporter cassette from the 4 *D4Z4* construct was transferred into the pBelo-BAC11 backbone and blocks of 4 additional *D4Z4* elements were added between the *eGFP* reporter and the telomere seed. Details are available upon request. The conditions of transfection of the linearized vectors with a modified calcium phosphate method [17] were optimized for each condition in order to obtain a single integration per cell. Three days post-transfection, the cells were treated with Hygromycin B (Roche Diagnostics) at a final concentration of 400µg/ml and kept under permanent selection. Harvested cells were analyzed using a FACScan flow cytometer and the data were processed using CellQuestPro software (Becton-Dickinson). The percentage of eGFP-positive cells was determined using the corresponding non-transfected cells as the baseline for autofluorescence. The mean values (M1) were also used to compare the fluorescence in the different samples. The integrity of each construct was determined by Q-PCR using primers for the *eGFP* sequence, *D4Z4* and a sequence encompassing the 3’ end of the vector backbone (Fig. S1D).

Pools of siRNA against Lamin B (*LMNB*, L-005270-00); *BANF1* (M-011536), *YY1* (M-011796-01), *USF1* (M-003618-00) and *USF2* (M-003617-00) were purchased from Dharmacon and transfected according to the manufacturer’s instruction using the Dharmafect 1 reagent.

**Formaldehyde cross-linking and chromatin immunoprecipitation.** *In vivo* protein-DNA cross-linking was carried out as described [24]. Generally, 1.5-2 x 10^8^ cells were harvested and proteins were then cross-linked to DNA for 10 min at room temperature followed by 40 min at 4°C with a final concentration of 1% formaldehyde in 0.1M NaCl, 1mM EDTA, 0.5 mM EGTA, 50 mM Tris pH 8. After lysis in the presence of SDS, nucleoprotein complexes were sonicated to reduce DNA fragments to 400-600 bp using a Bioruptor sonifier (Diagenode). To reduce non-specific background, the chromatin solution was precleared with salmon sperm DNA/protein A-agarose beads for 1h at 4°C. At this point, a fraction of DNA was prepared and further used as the input sample. Antibodies specific for Lamin A/C (Santa Cruz, Ref Sc 6215) or CTCF (Millipore, Ref 07-729) were incubated with protein A-clarified chromatin overnight at 4°C with gentle rocking. As controls, rabbit IgG (Sigma) were used for non-specific immunoprecipitation, and polyclonal anti H3 (Abcam Ref 1791) for the normalization of the immunoprecipitation. After immunoprecipitation, immune complexes were collected by adding 60 ml of salmon sperm DNA/protein A or G-agarose beads for 1h at 4°C. The bound and unbound chromatin were separated and the beads were washed, then complexes were eluted in 1% SDS, 0.1M NaHCO_3_ and cross-links were reversed by heating. DNA was recovered by proteinase K digestion, phenol extraction and ethanol precipitation. DNA samples were quantified using the NanoDrop ND-1000 spectrophotometer (NanoDrop technologies).

**Real time Q-PCR.** Q-PCR experiments were performed with a LightCycler system and using the Fast Start DNA Master SYBR Green I mix (Roche Diagnostics) according to the manufacturer’s instructions. Each amplification was carried-out in duplicate with 0.5 mM of sense and antisense primers and 10 ng of diluted DNA. Reaction mixtures were incubated for 8 min at 95 °C, followed by 45 cycles of 15s at 95°C, 4s at 61°C and 8s at 72°C. Fluorescence was acquired during each elongation step. The Ct is the number of PCR cycles necessary to reach a pre-determined fluorescence intensity and is a function of the amount of target DNA in the samples analyzed. For ChIP experiments, the fold-enrichment of target sequence from antibody-bound chromatin DNA (IP) compared to input (Ref) was calculated using the equation (2^Ct(IP)-Ct(Ref)^). Values were standardized to the H4 promoter and to the enrichment determined in the C33A control cells. Primer sequences are given in the **Supplementary Table 1**

# Total RNA extraction. Total RNA was extracted from cell lines using the RNeasy kit (Qiagen) and treated with *DNase*I following the manufacturer’s instructions. After extraction, the integrity of total RNA was examined on a 1.2% agarose gel containing 1 µg/ml ethidium bromide. RNA concentration was measured at 260 nm. The purity of the total RNA extracted was determined by the 260 nm/280 nm ratio with values between 1.8 and 2 and RNA samples were stored at -20°C until use.

**cDNA synthesis & Quantitative PCR.** For reverse transcription, cDNA was generated using 0.5 µg of total RNA, oligo(dT) primer, RNaseOUT (Recombinant Ribonuclease Inhibitor), and SuperScript II Reverse Transcriptase according to the manufacturer’s instructions (Invitrogen) in a total volume of 20 µl. Real-time quantitative PCR (RT-PCR) experiments were performed on 2.5 ng cDNA as mentioned above. The following amplification program was used: after 8 minutes of denaturation at 95°C, 45 cycles of real-time PCR with a 3-segment amplification were performed. Each cycle consists of a denaturation step of 15s at 95°C for, a 4s annealing at 61°C, and a 8s elongation at 72°C. The melting step was then performed with slow heating starting at 70°C with a rate of 0.1°C per second up to 98°C with continuous measurement of fluorescence. The quantification of the target genes was always normalized to the amount of β-actin cDNA in the samples.

**Constructs & enhancer blocking assay.**

The *D4Z4* element was cloned into the pNI vector [19] before or after removal of the distal *5’HS2 β-globin enhancer*. Details are available upon request. Enhancer blocking assays were performed as previously described [19]. Briefly, 200 ng of each construct linearized at the *Sal*I site were electroporated at 200 V and 960 µF into 1 x 10^7^ K562 cells. After 24 hours of recovery, cells were plated in soft agar with geneticin (G 418) at 750 µg/ml. Colonies were counted after 2-3 weeks of selection and the colony number was normalized to that obtained with pNI.

**Preparation of nuclear extracts and gel mobility shift assay.** Experiments were performed as described previously [23,24]. The following sequences were tested for CTCF binding, 468-L, AGA GGG GCG GAA GGG ACG TTA GGC AGG GAG GCA GGG AGG CAG GGA GGC AGG GAG GAA CGG AGG GAG; 468-S, GAC GTT AGG ACG GGA GGC AGG GAG GCA GGG AGG CAG G.

**Table 1.** **Primers used for quantitative real time PCR of the ChIP experiments.**

| **Name** | **Sequence** |
| --- | --- |
| **1-forward** | **5’ GGAGAGAGGAACGGGAGAGA 3’** |
| **1-reverse** | **5’ GGACGCTGACCGTTTTCC 3’** |
| **2-forward^1^** | **5’ ACGACGGAGG CGTGATTT 3’** |
| **2-reverse^1^** | **5’ AGTGTGGCCG GTTTGGAA 3’** |
| **3-forward^1^** | **5’ GGGCTCACCGCCATTCAT 3’** |
| **3-reverse^1^** | **5’ TGCACCTCAGCCGGACTGT 3’** |
| **4-forward^2^** | **5’ CTAGCGAGGA AGAATACCG 3’** |
| **4-reverse^2^** | **5’ ACCGGGCCTA GACCTAGAAG 3’** |
| ***eGFP*-forward** | **5’ AGGAGCGCACCATCTTCTT 3’** |
| ***eGFP*-reverse** | **5’ GATGTTGTGGCGGATCTTG 3’** |
| ***H4*-promoter forward** | **5’ TGTGTGATGGGAAGATGGGA 3’** |
| ***H4*-promoter reverse** | **5’ GAGCAAAATAGCGAGACCCC 3’** |

To minimize experimental variations, primers (Eurogentec) were designed with similar melting temperatures (61°C). The efficiency of each set of primers was determined from the slope of the linear regression equation and was validated for values ranging from 83 to 110% (-3.8/-3.1). Primers ^1^ were described in Jiang *et al*. [42]. Primers ^2^ were described in Espada *et al*. [49]. Primers for chromosome 6, 7 and 20 are described in Wendt *et al* [25].

**Table 2.** **Primers used for quantitative real time PCR of the siRNA experiments.**

|  |  |
| --- | --- |
| **Name** | **Sequence** |
| **CTCF-Forward^3^** | **5’ gaacccattcaggggaaaagc 3’** |
| **CTCF-Reverse^3^** | **5’ tcgcaagtggacacccaaatc 3’** |
| **LMNA-Forward** | **5’ gacccatctcctctggctct 3’** |
| **LMNA-reverse** | **5’ ctggcaggtcccagattaca 3’** |
| **β-actin-Forward** | **5’ tccctggagaagagctacga 3’** |
| **β-actin-Reverse** | **5’ agcactgtgttggcgtacag 3’** |

Primers ^3^ were described in Loukinov *et al*.[50].
